# Sciatica of Twelve Years' Standing Cured by Acupuncture

**Published:** 1831-04-01

**Authors:** 


### IV. Sciatica of Twelve Years' Standing cured by Acupuncture.

A case of this kind is reported from the Western Dispensary: The patient was an aged female, and the pain was almost intolerable. Colchicum, Dover's powder, and various other means, having proved unprofitable, acupuncture was employed and the pain instantly ceased. There was no return of the sciatica!

